# Antifungal activity of pomegranate peel extract and isolated compound punicalagin against dermatophytes

**DOI:** 10.1186/s12941-014-0032-6

**Published:** 2014-09-05

**Authors:** Simone R Foss, Celso V Nakamura, Tania Ueda-Nakamura, Diógenes AG Cortez, Eliana H Endo, Benedito P Dias Filho

**Affiliations:** Post graduation in Microbiology, Universidade Estadual de Londrina, Londrina, PR Brazil; Department of Health Sciences, Universidade Estadual de Maringá, Maringá, PR Brazil; Departament of Pharmacy, Universidade Estadual de Maringá, Maringá, PR Brazil; Post graduation in Pharmaceutical Sciences, Universidade Estadual de Maringá, Maringá, PR Brazil

## Abstract

**Background:**

Dermatophyte species infect the epidermis and appendages, often with serious social and health-economic consequences. The hydroalcoholic extract of pomegranate fruit peel showed activity against the dermatophyte fungi *Trichophyton mentagrophytes, T. rubrum, Microsporum canis* and *M. gypseum.*

**Methods:**

Hydroalcoholic extract was prepared with pomegranate peels. This crude extract was fractionated and submitted to liquid-liquid partition, resulting in an active fraction which was fractionated in a Sephadex LH-20 column, followed by a Lobar column. The structure of the active compound was established with the use of spectroscopic methods.

**Results:**

The crude extract of pomegranate fruit peel showed activity against the dermatophytes *Trichophyton mentagrophytes, T. rubrum, Microsporum canis,* and *M. gypseum,* with MICs values of 125 μg/ml and 250 μg/ml, respectively for each genus. Punicalagin was isolated and identified by spectroscopic analysis. The crude extract and punicalagin showed activity against the conidial and hyphal stages of the fungi. The cytotoxicity assay showed selectivity for fungal cells than for mammalian cells.

**Conclusions:**

These results indicated that the crude extract and punicalagin had a greater antifungal activity against *T. rubrum*, indicating that the pomegranate is a good target for study to obtain a new antidermatophyte medicine.

## Background

Dermatophytes are fungi that use keratin for their nutrition and may cause infections of the nails, skin, and hair, known as dermatophytosis. These organisms are classified into three genera: *Epidermophyton, Trichophyton,* and *Microsporum* [1]. Although not life-threatening, superficial mycoses due to dermatophytes have been among the most common communicable diseases of humans since antiquity, and have considerable social and health-economic implications [2].

Generally, dermatophytes infect the superficial layers of skin. However, immunocompromised patients, such as AIDS patients or recipients of kidney transplants, can be affected by deep injury in the dermal layer, resulting in disseminated lesions that may take fatal forms [3-6]. Although many antifungals are available, their side effects and drug interactions, and the existence of resistant organisms have created a need to find safer and more effective treatments [7]. Also, dermatophytosis treatments are, in general, expensive and must be applied over long periods.

Natural products have proven to be an alternative source of new active molecules. In many countries, mainly in developing countries, plants have been used as the primary basic health treatment. The pomegranate *Punica granatum* is a bush 3 to 5 meters in height, with opposite and obtuse leaves, flowers with wrinkled white, yellow, or orange petals. The fruit is composed of a yellow to red peel that covers the seeds, the fleshy arils of which are eaten. *Punica granatum* is a plant with worldwide application in folk medicine. There are references to an antimicrobial effect of pomegranate products against many pathogenic bacterial species, including inhibition of formation of biofilms [8-11], antiplasmodial activity, and effects against *Entamoeba histolytica* and *Giardia lamblia* [12]. Polyphenols extracted from pomegranate fruit rind were active against phytopathogenic fungi [13]. The extract of *P. granatum* showed good results as a topical antifungal agent for the treatment of candidosis associated with denture stomatitis [14]. The tannin punicalagin is the major component of pomegranate fruit peel. This substance was isolated not only from *Punica granatum,* but also was described from *Terminallia mollis* and *Terminallia brachystemma,* as having antifungal activity against *Candida albicans, C. krusei,* and *C. parapsilosis* [15].

The present study evaluate the antidermatophyte activity of pomegranate fruit peel extract and investigate its effect on different fungal development stages, cytotoxicity and possible mechanisms of action. The active substance of pomegranate peel was isolated and identified as well.

## Methods

### Plant material and crude extract

*Punica granatum* fruits were collected in December 2007 in Maringá, Paraná, Brazil. The peel was separated manually (2183.8 g) and extracted with a 90% (v/v) hydroalcoholic solution, by maceration at room temperature for 5 days in a dark room. The hydroalcoholic extracts were filtered, evaporated under vacuum at 40°C, lyophilized, and kept in a freezer at −10°C. This crude extract was assayed against four species of dermatophyte fungi and Gram-positive and Gram-negative bacteria.

### Isolation of the active substance

First, 200 ml of an aqueous solution of the crude extract (20 g) was submitted to liquid-liquid partition, and eluted with ethyl acetate and then with *n*-butanol; this procedure was repeatead four times with each solvent, resulting in three fractions: F1 (water), F2 (ethyl acetate), and F3 (*n-*butanol). The collected fractions were evaporated under vacuum and lyophilized in the same conditions as for the extract. Second, 0.5 g of the fraction with the best activity (F1) was dissolved in water, filtered through cotton wool and then placed in a Sephadex LH-20 column. The procedure was performed twice to maximize the yield. It was monitored by thin-layer chromatography (TLC), mobile phase *n-*butanol: acetic acid: water (40:10:50), and observed as a natural yellow substance. Finally, after antifungal tests, the active subfraction was placed in a Lobar (C-18) column and eluted with methanol:water (1:1), also monitored by TLC. The structure of the active compound was established with the use of spectroscopic methods (EI-MS, ^1^H NMR, ^13^C NMR, H-H COSY, HMBC, HMQC, and DEPT). The isolated substance was tested against *Trichophyton rubrum.*

### Microorganisms and growth conditions

Dermatophyte species used for this investigation were *Microsporum canis* ATCC 32903*, Microsporum gypseum* ATCC 14683*, Trichophyton mentagrophytes* ATCC 1481 and *Trichophyton rubrum* ATCC 28189. Gram-negative bacteria *Pseudomonas aeruginosa* ATCC 27853 and *Escherichia coli* ATCC 25922 and Gram-positive bacteria *Bacillus subtilis* ATCC 6623 and *Staphylococcus aureus* ATCC 25923 were also investigated, because secondary infections may occur in dermatophytoses.

### Microdilution broth assay

Antifungal and antibacterial assays were performed by microdilution method in sterile flat-bottom microplates according to CLSI [16,17]. Each well contained appropriate test samples, culture medium, and approximately 10^5^ cells for bacteria, and 10^4^ spores in a total volume of 100 μl. The plates were incubated at 37°C and 24 h for bacteria and 28°C during 72 h for dermatophytes. The MIC (Minimal Inhibitory Concentration) was defined as the lowest concentration of a compound at which the microorganism tested did not demonstrate visible growth. To determine the minimal fungicidal effect, 10 μl of suspension from the MIC was spotted in Sabouraud agar and incubated for 24 to 72 h at 28°C. The minimum fungicidal concentration (MFC) was defined as the lowest concentration that yielded negative subcultures or only one colony.

### Conidial germination inhibition assay

Different concentrations of test samples in 90 μl were prepared in 96-well flat-bottom micro-culture plates by the double dilution method. The wells were prepared in duplicate for each concentration. An inoculum of 10 μl of spore suspension containing 2000–3000 spores was added to each well. Plates were incubated at 28°C for 24 h and then examined for spore germination under an inverted microscope. For analysis, spores were considered germinated if they had a germ tube at least twice the length of the spore.

### Disc diffusion method

Disc diffusion method and fluorescence microscopy were used to evaluate the hyphal growth inhibition. Plates with Sabouraud Dextrose Agar were centrally inoculated with *T. rubrum* and incubated at 28°C for 3–5 days. Test discs were made with the extract, punicalagin, and Nystatin, with concentrations close to the MIC. These discs and one control disc (with 10 μl of sterile water) were arranged around the colony on the plate, at a distance of 0.5 cm, and incubated at 28°C for 72 h. The hyphal growth inhibition was evaluated visually and photographed [18].

### Fluorescence microscopy

Sub-inhibitory concentrations of the crude extract in 500 μl of culture medium were prepared by the double-dilution method, in 24-well flat-bottom micro-culture plates, on which round cover slips were placed. The wells were prepared in duplicate for each concentration. The wells were inoculated with 50 μl of spore suspension, containing 10,000–15,000 spores. The plates were incubated at 28°C for 24 h. Then, the cover slips carefully removed were and washed in PBS, pH 7.2, with light manual shaking. Next, the medium was carefully removed and cover slips with adhered cells were stained with Calcofluor White M2R (Sigma, St. Louis, MO, USA) and mounted on a slide with synthetic resin (Araldite 502™). Slides were viewed by means of a Zeiss fluorescent microscope [19].

### Cytotoxicity assay

Confluent Vero cell monolayers grown in 96-well cell-culture plates were incubated with a tenfold serial dilution of punicalagin, starting with a concentration of 1000 μg/ml - for 48 h at 37°C and 5% CO_2_. At that time, cultures fixed with 10% trichloroacetic acid for 1 h at 4°C were stained for 30 min with 0.4% sulforhodamine B (SRB) in 1% acetic acid and subsequently washed with distilled water. Bound SRB was solubilized with 150 ml 10 mM Tris-base solution. Absorbance was read in an ELISA plate reader at 530 nm. The cytotoxicity was expressed as a percentage of the optical density compared to the control.

## Results and discussion

### Isolation of active substance

From 2183.8 g of pomegranate fruit peel, 220.6 g of crude extract was obtained, a yield of 10.1%. Pomegranate fruit peel is rich in tannins, high-molecular-weight plant polyphenols, which can be classified into two chemically and biologically distinct groups: condensed tannin and hydrolyzable tannin, the latter composed of phenolic acids and glycosyl esters. Hydrolyzable tannins are separated into ellagitannins (containing ellagic acid) and gallotannins (containing gallic acid) [20]. The structure of active compound, punicalagin, obtained by successive bioactive-guided steps, was established by spectroscopic methods (EI-MS, ^1^H NMR, ^13^C NMR, H-H COSY, HMBC, HMQC, and DEPT). Punicalagin has been isolated from plants [21] by different methods. However, some of the isolation techniques, such as high-speed countercurrent chromatography, require expensive apparatus [22]. The present method is a less expensive alternative to obtain punicalagin with excellent results, and resembles a purification procedure reported [23]. The spectral analysis was compared with that reported by Doig *et al*. [24], which confirmed the isolated substance as punicalagin. The mass spectrum (EI-MS) of the punicalagin isolated confirmed to that reported by Seeram *et al*. [23]. Punicalagin anomers can be observed in the mass spectrum, which show double chemical shifts at the same carbon or hydrogen.

Punicalagin FAB-MS at *m/z* 1083.4 found C_48_H_28_O_30_. The ^13^C NMR results: δ 88.78 (C-1), 69.73 (C-2), 75.33 (C-3), 73.13 (C-4), 65.38 (C-5), 63.23 (C-6), 168.26 (C-7), 124.59 (C-8), 105.61/108.71 (C-9), 144.72 (C-10), 135.67 (C-11), 144.72 (C-12), 114.42 (C-13), 113.87 (C-14), 144.62 (C-15), 135.5 (C-16), 144.4 (C-17), 105.02/108.71 (C-18), 124.24 (C-19),168.45/148.57 (C-20), 167.6 (C-21), 123.57 (C-22), 109.51 (C-23), 144.39 (C-24), 137.76 (C-25), 144.39 (C-26), 117.7 (C-27), 109.51 (C-28), 147.35 (C-29), 136.86 (C-30), 135.5 (C-31),112/113.37 (C-32), 121.75/121.87 (C-33), 157.81 (C-34), 112.56/113.37 (C-35), 135.18 (C-36),136.86/139.2 (C-37), 147.35 (C-38), 109.51 (C-39), 121.75/121.87 (C-40), 157.3 (C-41),113.87 (C-42), 144.36 (C-43), 134.88 (C-44), 144.11 (C-45), 105.02/108.71 (C-46), 124.59 (C-47), 168.20 (C-48). HMBC^1^H-^13^C and ^1^H results: 5.06/5.13 (H-C1, *m*), 4.65/4.71 (H-C2, *m*),5.06/5.13 (H-C3, *m*), 4.65/4.71 (H-C4, *m*), 3.09/3.17 (H-C5, *m*), 4.03 (Hα-C6, *m*), 1.83 (Hβ-C6,*d*), 6.39/6.41 (H-C9, *s*), 6.28/6.41 (H-C18, *s*), 6.7 (H-C23, *s*), 6.28/6.41 (H-C46, *s*).

### Antifungal effect

Crude extract from pomegranate showed a considerable inhibitory effect against both genera *Trichophyton* and *Microsporum* (Table 1) with MIC of 125 and 250 μg/ml, respectively. Results of specific tests against *T. rubrum* are presented in Table 2. The isolated compound punicalagin showed about the same MIC value as the crude extract, probably because punicalagin is the majority substance. Nystatin showed MIC of 0.39 μg/ml for all dermatophytes tested. The minimal fungicidal concentration of the crude extract against *T. rubrum* was within two-twofold dilution of the MIC for this organism. Plant products tested for use against dermatophytes have shown activity against *T. rubrum* [19,25,26]. Although the mechanisms of action were not elucidated, we eliminated the possibility of complexation with the membrane ergosterol (data not shown) and observed no change in the morphology of the hyphal structures.

**Table 1:** **Minimal inhibitory concentration (MIC)**

**Microorganisms**	**MIC (μg/ml)**
**Bacteria**	
*Staphylococcus aureus*	62.5
*Bacillus subtilis*	62.5
*Pseudomonas aeruginosa*	250
*Escherichia coli*	>1000
**Dermatophytes**	
*Trichophyton rubrum*	125
*Trichophyton mentagrophytes*	125
*Microsporum gypseum*	250
*Microsporum canis*	250

Minimal inhibitory concentration (MIC) of crude extract of *Punica granatum* against bacteria and dermatophytes species.

**Table 2:** **Antifungal activity (μg/ml) against** ***Trichophyton rubrum***

	**Antifungal activity (μg/ml) against** ***Trichophyton rubrum***		
	**MIC**	**MFC**	**CGI**
Crude extract	125	250	62.5
Fractions			
P1	125	125	62.5
P2	250	250	-
P3	125	125	-
Punicalagin	62.5	125	62.5
Nystatin	0,78	-	0,39

Minimal inhibitory concentration (MIC), minimal fungicidal concentration (MFC) and conidal germination inhibition (CGI) of pomegranate fruit peel extract, fractions and the isolated substance punicalagin.

Under certain conditions, dermatophytosis can be complicated by secondary bacterial infections. Therefore, we investigated whether the hydroalcoholic extract exerts, in addition to its antifungal effects, a significant antibacterial activity against Gram-negative and Gram-positive bacteria. The crude extract from pomegranate showed good activity on *S. aureus, B. subtilis* and *P. aeruginosa* with MICs of 62.5, 62.5 and 250 μg/ml. *E. coli* MIC >1000 μg/ml were considered resistant (Table 1).

### Conidial germination

There are two phases of fungal growth, conidial germination and hyphal growth, in which drug action can occur. Conidial germination inhibition occurred at concentration of 62.5 μg/ml for both crude extract and punicalagin. Nystatin was able to inhibit conidial germination at 0.39 μg/ml. This is particularly noteworthy because the MICs of crude extract and fraction extract were found to be 125 μg/ml. This similar effect of the extract and isolated substance is due to the fact that punicalagin is the majority substance in the pomegranate fruit peel [22].

### Disc diffusion

**Figure 1:**
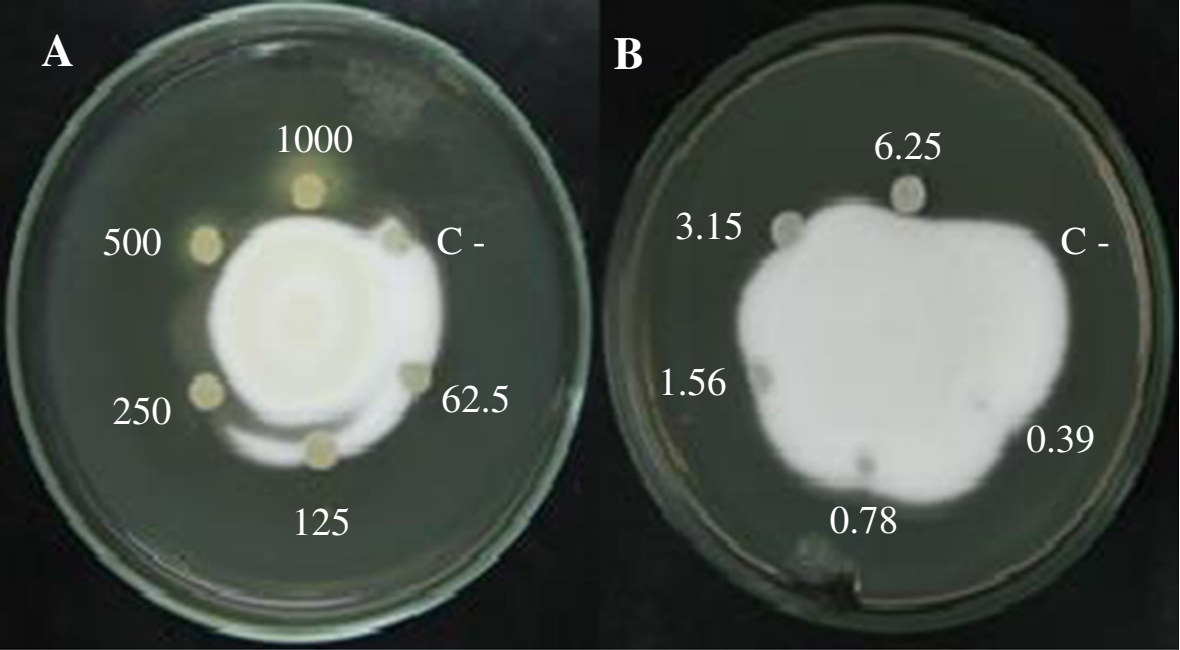
**Disc diffusion method** ***.*** Antifungal activity in solid medium against *T. rubrum*. **(** **A** **)** Crude extract – 1000, 500, 250, 125, 62.5 μg/ml. **(** **B** **)** Nystatin – 6.25, 3.15, 1.56, 0.78, 0.39 μg/ml. Water was used as control (C-). Data correspond to one representative experiment out of three.

Disc diffusion is simple and inexpensive agar-based method which enables the determination of activity of different substances against microrganisms. Here, the hyphal growth inhibition is shown with disc diffusion of crude extract and Nystatin in agar. In Figure 1, discs containing at least 250 μg/ml of crude extract inhibited hyphal growth of *T. rubrum*.

### Fluorescence microscopy

**Figure 2:**
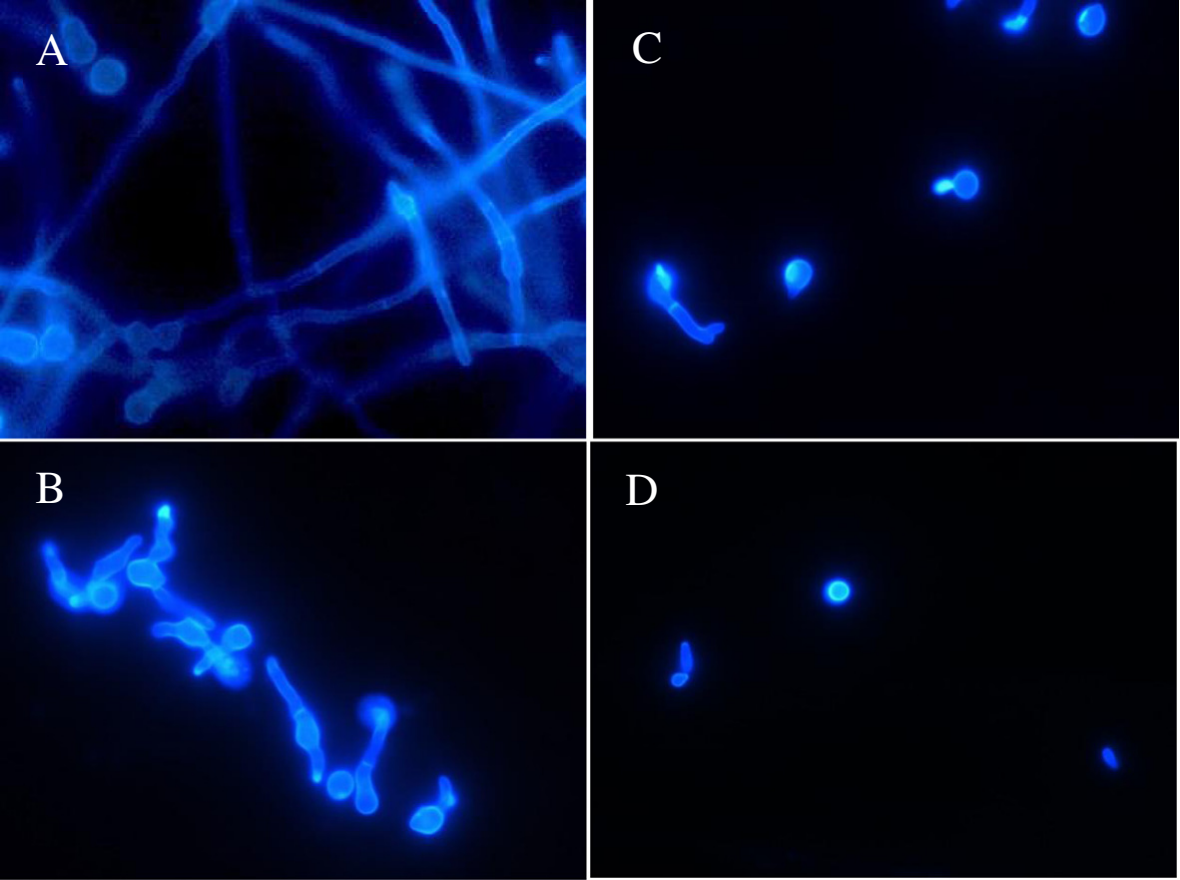
**Fluorescence microscopy by Calcofluor White Stain.** Spore germination inhibition of *T. rubrum* on cover slips. **(** **A** **)** Control cells; **(** **B** **)** Treated with 125 μg/ml crude extract; **(** **C** **)** Treated with 62.5 μg/ml punicalagin; **(** **D** **)** Treated with 0.78 μg/ml Nystatin. Data correspond to one representative experiment out of three.

Calcofluor White stain was used to show possible fungal cell wall alterations. Figure 2 shows strong inhibition of hyphal growth on *T. rubrum* treated with crude extract at 125 μg/ml (Figure 2B) and inhibition of conidial germination by punicalagin at 62.5 μg/ml (Figure 2C). Although no morphological alterations were detected, this procedure was important to understand and confirm the inhibition of the hyphal growth and conidial germination, using the same incubation conditions. This may be explained by the nature of the principal substance, punicalagin, which is a tannin. The tannins could act on the microorganism cell membrane, switching its metabolism; complexing with metallic ions needed for the microorganism’s metabolism; and inhibiting fungal and bacterial enzymes by complexation with substrates [21].

### Cytotoxicity assay

Cytotoxicity was monitored using SRB assays. Cell viability after exposure to 100 μg/ml of crude extract, fraction P1, and punicalagin was 83%, 99%, and 90%, respectively (data not shown). The concentration of punicalagin with 50% cytotoxicity (CC_50_ value) on Vero cell was 400 μg/ml, showing that punicalagin is 6.4 times more selective for fungi cells than for mammal cells, indicating that the crude extract may be ideal for use in topical form. The values of CC_50_ of punicalagin are similar to those that have been reported, with CC_50_ as 460 μg/ml [21]. The acute toxicity evaluated, *in vivo,* using Wistar rats with intranasal administration, and detected that the LD_50_ (the dose that can kill 50% of the assayed animals) was 731 mg/ml [27]. Pomegranate juice may contain 2 g/L of punicalagin. Sprague–Dawley rats fed a diet containing 6% punicalagin, for 37 days, showed no toxicity [28]. These data support the initial idea of a treatment with crude extract of pomegranate fruit peel in the topical form.

## Conclusions

Pomegranate extract inhibited the growth of *T. rubrum*, *T. mentagrophytes*, *M. canis* and *M. gypseum*. Spectroscopic analyses revealed punicalagin as the active substance. The antidermatophyte assay, using *Trichophyton rubrum* as a model, showed that the crude extract acts on conidial and hyphal structures. Also, cytotoxicity assay showed that punicalagin was more selective for fungal than mammal cells, indicating its probable best use in clinical applications. These results indicated that the crude extract and punicalagin had a greater antifungal activity against *T. rubrum*, indicating that pomegranate is a good target for study due to its potential future use as a new therapeutic alternative against dermatophytosis.
